# Supplementary material for: Identification of photoperiod-induced specific miRNAs in the adrenal glands of Sunite sheep (Ovis aries)
Source: Front Vet Sci. 2022 Jul 22;9:888207. doi: 10.3389/fvets.2022.888207 (PMC9354845; doi:10.3389/fvets.2022.888207)
Supplement: Supplementary file 1 [file Data_Sheet_1.ZIP › Supplementary file/miRDeep2 file.zip!/miRDeep2 file/Novel_Structure/SPLP42A1/Novel_136.pdf]

Provisional ID	:	NC_019467.2_15272
Score total	:	4.6
Score for star read(s)	:	3.9
Score for read counts	:	-2.8
Score for mfe	:	2.1
Score for randfold	:	1.6
Score for cons. seed	:	
Total read count	:	6
Mature read count	:	4
Loop read count	:	0
Star read count	:	2

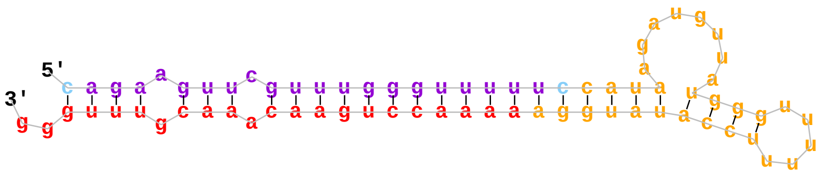

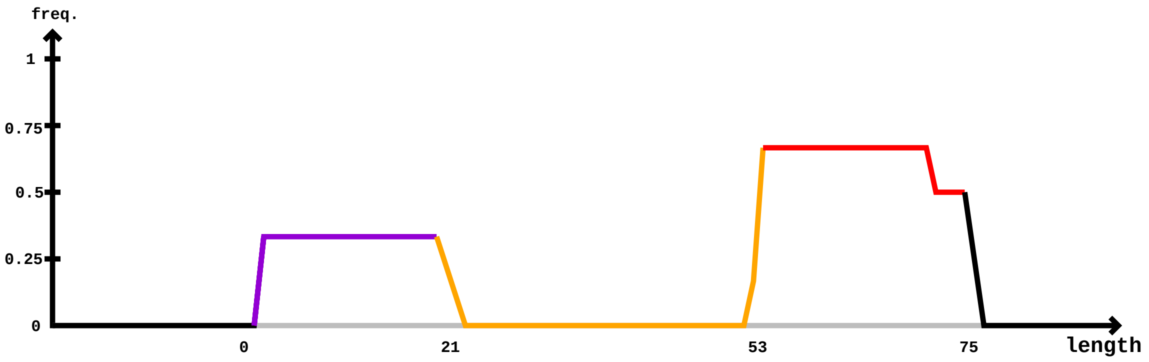


```
                      Star                                                Mature
5'- gucaucuguuggguuggcccagaaguucguuuggguuuuuccauaagauguuauggguuuuuuccauauggaaaaaccugaacaaacguuugggCcaaccaauacaacu   -3'    obs
    gucaucuguuggguuggcccagaaguucguuuggguuuuuccauaagauguuauggguuuuuuccauauggaaaaaccugaacaaacguuuggggcaaccaauacaacu          exp
    .......((((((((.((((((.(((.((((((((((((((((((........((((.....))))))))))))))))))))).))).)))))).)))))....)))).   reads     mm        sample
    ....................agaaguucguuuggguuuu......................................................................   1         0         seq
    ....................agaaguucguuuggguuuuu.....................................................................   1         0         seq
    .......................................................................aaaaaccugaacaaacUuu...................   1         1         seq
    ........................................................................aaaaccugaacaaacUuuuggg...............   3         1         seq
```
